# Research in action: using positive deviance to improve quality of health care

**DOI:** 10.1186/1748-5908-4-25

**Published:** 2009-05-08

**Authors:** Elizabeth H Bradley, Leslie A Curry, Shoba Ramanadhan, Laura Rowe, Ingrid M Nembhard, Harlan M Krumholz

**Affiliations:** 1 Division of Health Policy and Administration, School of Public Health, Yale University School of Medicine, New Haven, CT, USA; 2 Yale School of Management, New Haven, CT, USA; 3 Section of Cardiovascular Medicine and the Robert Wood Johnson Clinical Scholars Program, Department of Internal Medicine, Yale University School of Medicine; Center for Outcomes Research and Evaluation, Yale-New Haven Hospital, New Haven, CT, USA

## Abstract

**Background:**

Despite decades of efforts to improve quality of health care, poor performance persists in many aspects of care. Less than 1% of the enormous national investment in medical research is focused on improving health care delivery. Furthermore, when effective innovations in clinical care are discovered, uptake of these innovations is often delayed and incomplete. In this paper, we build on the established principle of 'positive deviance' to propose an approach to identifying practices that improve health care quality.

**Methods:**

We synthesize existing literature on positive deviance, describe major alternative approaches, propose benefits and limitations of a positive deviance approach for research directed toward improving quality of health care, and describe an application of this approach in improving hospital care for patients with acute myocardial infarction.

**Results:**

The positive deviance approach, as adapted for use in health care, presumes that the knowledge about 'what works' is available in existing organizations that demonstrate consistently exceptional performance. Steps in this approach: identify 'positive deviants,' *i.e*., organizations that consistently demonstrate exceptionally high performance in the area of interest (*e.g*., proper medication use, timeliness of care); study the organizations in-depth using qualitative methods to generate hypotheses about practices that allow organizations to achieve top performance; test hypotheses statistically in larger, representative samples of organizations; and work in partnership with key stakeholders, including potential adopters, to disseminate the evidence about newly characterized best practices. The approach is particularly appropriate in situations where organizations can be ranked reliably based on valid performance measures, where there is substantial natural variation in performance within an industry, when openness about practices to achieve exceptional performance exists, and where there is an engaged constituency to promote uptake of discovered practices.

**Conclusion:**

The identification and examination of health care organizations that demonstrate positive deviance provides an opportunity to characterize and disseminate strategies for improving quality.

## Introduction

Despite decades of efforts to improve quality of health care, poor performance persists in many aspects of care. Patients often do not receive guideline-recommended processes of care [1-3], and risk-adjusted outcomes vary substantially across hospitals [4] and regions [5,6], suggesting potential for improvements. Furthermore, despite enormous national investment in biomedical research, less than 1% of this is directed at research on improving health care delivery [7], and when innovations in clinical care are discovered, the uptake of these improvements into practice is often delayed and incomplete [8-11].

We describe an approach to quality of care research that identifies innovative strategies from 'positive deviants' in health care, those organizations that consistently demonstrate exceptionally high performance in an area of interest (*e.g*., survival rates, medication use, and timely emergency treatment). The central premise of a positive deviance approach [12,13] is that solutions to problems that face a community often exist within that community, and that certain members possess wisdom that can be generalized to improve the performance of other members. Many of these strategies rely on resources that already exist in the community, which can increase their adoption and sustained use [14].

The power of a positive deviance approach to improve health outcomes has been shown in complex problems globally, including pregnancy outcomes [15], condom use [16], and childhood nutrition [12,17,18]. In a dramatic application of positive deviance in Vietnam, childhood malnutrition was reduced by 75% [12]. Researchers identified a set of women as 'positive deviants' because their children were thriving despite high rates of childhood wasting and stunting in their rural villages. The women were including in their cooking pots tiny shrimps and crabs, found in large numbers in rice paddies but not normally used because fish were generally thought to be inappropriate for young children [18]. The subsequent randomized controlled trial showed significant improvements in health outcomes of children fed in this way [12,17,19]. This method of food preparation was then disseminated and sustained years after the original studies [20]. The 'best practice' was based on proven, successful practices within the community, rather than theoretical concepts of good nutrition.

How might this potentially powerful approach be used to improve quality of health care delivery in the United States? How does it differ from other strategies of identifying and disseminating best practices, and in what circumstances might this approach be most effective? We address these questions in the following five sections. In the first section, we provide an overview of the positive deviance approach as applied to the organizational setting and discuss when its application is most useful. In the second section, we outline core methodological considerations in this approach. In the third section, we compare the positive deviance approach to alternative methods of identifying best practices, including standard biomedical and epidemiologic research and quality improvement and action research. In the fourth section, we draw on theoretical literature to describe how the positive deviance approach can promote effective dissemination of best practices. We conclude with an illustrative example of the positive deviance approach applied to improving hospital care nationally for patients with myocardial infarction.

### Overview of positive deviance approach

**Figure 1:**
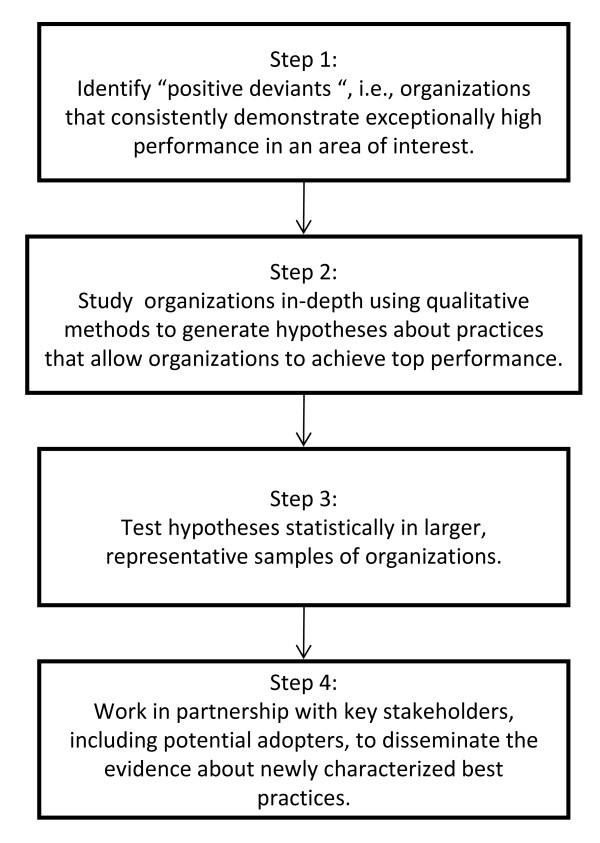
**Steps in the positive deviance approach**.

The positive deviance approach accomplishes two goals: the identification of practices that are associated with top performance, and promoting the uptake of these practices within an industry, using the following steps (Figure 1): identify 'positive deviants,' *i.e*., organizations that consistently demonstrate exceptionally high performance in the area of interest (*e.g*., proper medication use, timeliness of care); study the organizations in-depth using qualitative methods to generate hypotheses about practices that enable organizations to achieve top performance; test hypotheses statistically in larger, representative samples of organizations; and work in partnership with key stakeholders, including potential adopters, to disseminate the evidence about newly characterized best practices.

When should one consider using a positive deviance approach to identify and disseminate best practices in health care organizations? First, the approach requires concrete, widely endorsed, and accessible performance measures for organizations. For instance, in the case of hospital care, there are several specific, validated, and publicly-reported performance measures; therefore, hospitals can be ranked according to performance, and positive deviants within the industry can be identified. In contrast, there are no publicly accessible data on performance measures for many health care conditions such as treatment of children with fevers or hospital falls among elderly, among others. Positive deviance studies in these areas would therefore be difficult to accomplish.

Second, the positive deviance approach works when there is variation in organizational performance and outcomes across the industry, with some organizations achieving marked and consistent top performance and other organizations not doing so, *i.e*., there are positive deviants. Additionally, the approach is effective when organizations are adequately open to sharing their strategies for exceptional performance. In cases where organizations are highly proprietary and resistant to sharing what might be viewed as competitive advantages or 'trade secrets,' the positive deviance approach is unlikely to produce meaningful results.

Third, the approach is effective when hypotheses generated from the experience of top performing organizations can be tested in larger, representative samples. Evidence from statistical testing is particularly useful when disseminating findings to health care organizations because clinicians, whose support is often fundamental to successful changes in clinical processes [21,22], are more likely to consider such evidence credible and valid.

Finally, for potential adopting organizations, the perceived importance of improvement on the selected performance measure can enhance effective dissemination. Involving potential adopters in the development and testing of a particular practice can also accelerate the pace and scope of uptake by increasing the fit of the practice with the organizational context.

### Methodological considerations in the positive deviance approach

#### Sampling strategy and sample size

Studies using positive deviance begin with purposive sampling, with the goal of selecting organizations based on diversity of performance with adequate representation of organizations with exceptional performance. As is standard in purposive sampling for qualitative studies [23], the sample should be diverse in characteristics potentially salient to performance, such as size, ownership type, teaching status in the case of hospitals, and geographical location. Ensuring adequate diversity among the top performing organizations studied is critical to isolating through several cases what might be common in achieving top performance, as well as enhancing the transferability of findings to a broad range of potential adopters. Principles of qualitative research are used to develop the sampling strategy [23], and the sample size at this first stage is determined by theoretical saturation [24], *i.e*., when successive sampling does not produce additional hypotheses.

The sampling strategy for the next stage of a positive deviance study, in which one is statistically testing hypotheses generated from the qualitative study, employs methods for quantitative investigation. The goal is to sample the universe of relevant organizations in order to attain a large, representative sample of the industry to which one is generalizing, thereby permitting valid and precise inferences from subsequent statistical analysis. Sample size is determined by considerations of statistical power and desired level of precision.

#### Data collection and measurement

The in-depth examinations of organizations requires open-ended, qualitative data collection methods that explore both specific strategies taken by organizations as well as the broader context in which such strategies are employed [23,25]. A particular benefit of the positive deviance approach is the ability to integrate organizational context (*e.g*., concepts of organizational culture, norms of behavior, inter-group relations) into the understanding of 'what works' or best practices. This integration is often neglected in randomized controlled trials and difficult to measure in quantitative studies. Data collection may include observations, in-depth interviews and focus groups with staff, archival reviews of documents from the organization, or a combination of these methods, with the goal of developing a deep understanding of the organization and how it functions relative to the particular performance measures.

A core challenge and opportunity in positive deviance studies is the linking of the qualitative findings (*i.e*., hypotheses) and the quantitative measures of those variables hypothesized to influence performance. A benefit of the mixed methods approach [26], when qualitative precedes quantitative studies, is the richness of information that can then inform the development of comprehensive and precise quantitative measurement. At the same time, some hypotheses may include constructs for which there are not validated quantitative measures or for which quantitative measures cannot be developed. In such cases, it is not uncommon to restrict the statistical measurement to those hypotheses that lend themselves to quantitative measurement, recognizing that the best practices may ultimately emerge from the union of findings from both studies.

#### Data analysis

Data analysis should be conducted in accordance with standard principles for qualitative and for quantitative analysis [23,27]; however, it is of critical importance that the 'outcome' variable is well-measured with precision and validity, as it not only determines the initial purposeful sample but also forms the bases of the outcome measurement for the quantitative study. The performance measure(s) should be well-conceived and widely endorsed prior to the study.

#### Comparisons of alternative approaches

How does the positive deviance approach compare with other approaches to identifying and disseminating best practices? Although there are many, we focus on two alternative approaches to the identification of best practices, which are commonplace in research on quality of care: biomedical or epidemiological outcomes research, and quality improvement [28,29] and action research [30-34]. We discuss the theoretical underpinnings of these approaches, comparing and contrasting them to the positive deviance approach.

#### Biomedical or epidemiologic outcome research approaches

Biomedical or epidemiologic outcomes research focuses on developing an evidence base through quantitative measurement and statistical examination of a variety of predictors or correlates of an identified outcome, *i.e*., a performance measure. In health care, for instance, hierarchical generalized linear models [35,36] can be used to estimate hospital-level effects from patient-level data to isolate what might be organization-level variables (*i.e*., clinical protocols, data audit and feedback processes) that are statistically related to an outcome (*i.e*., complication rates, timeliness of care).

The advantage of this approach to identifying best practices is that the production of statistical associations is often based on the experience of a large sample of organizations and, particularly for health care, produced in a language and with methods that are credible to physicians whose involvement is often important for successful adoption and implementation of best practices by a health care organization.

Conversely, a disadvantage of this approach is that it typically neglects the complexity of organizational context, which is problematic given that organizational factors can be important barriers to implementation of innovative practices or programs [37-39]. Randomized or controlled trials standardize implementation procedures, limiting the understanding of how real-life variation in implementation (*e.g*., differences in monitoring functions, reward systems, leadership styles) might influence the impact of various practices on the outcome. Furthermore, such studies do not delve into the variation within the intervention or non-interventions arms of the trials to understand how organizational context might influence the success of the intervention. As a result, while such trials produce useful data, they do not provide insight into organizational features such as inter-group relations, leadership, and culture might influence the impact of the intervention on performance. Furthermore, the organizations in which such studies are conducted may be systematically different from most. Although this is the concern of generalizability from any type of research, organizations that participate in randomized and controlled trials may be particularly distinct (often large teaching or research facilities) from potential adopting organizations. In summary, such studies can provide credible statistical evidence, particularly if they are integrated in the hypothesis testing step of positive deviance studies; however, used in isolation, such studies they may oversimplify recommendations for best practices with inadequate attention to the subtleties of implementation, thereby slowing their translation into practice and widespread uptake.

#### Quality improvement and action research approaches

Quality improvement and action research, as applied to organizations, both focus on developing best practices within focal organizations. The approaches recognize the importance of organizational context, and the goal of developing best practices for the selected organization. Quality improvement [28,40] seeks to improve and/or reduce variation in work processes to improve the organization's ability to meet its goals. Action research, as applied to organizations, uses an iterative cycle of problem identification, planning, intervention, and evaluation to develop innovative solutions through researcher-staff collaboration in problem solving [30-32,41]. In both quality improvement and action research, the emphasis is on internal development and implementation of best practices for that particular organization or unit within the organization.

There are strengths to these approaches, which have been shown to improve targeted administrative and clinical performance measures in health care [28,42]. For example, substantial organizational learning can arise from quality improvement and action research projects; such learning can ultimately improve the identified process as well as provide staff expertise and create norms that allow staff to subsequently improve other processes in the organization. In addition, the approaches do recognize the importance of organizational context, building knowledge about 'what works' within the context of the internal organization, and potentially thereby improving success in implementation within that organization.

However, there are also important limitations to consider. The process of development of best practices in these approaches is informed typically by a very small sample of organizations, even a single organization or unit within an organization. Particularly for action research, solutions are developed within and for a selected organization; these solutions may not be amenable to widespread dissemination, thus limiting opportunities for large-scale change. In addition, these approaches neglect potential extant knowledge among other organizations that have previously attained top performance, which is not integrated into the quality improvement or action research efforts. Finally, neither quality improvement nor action research has an explicit goal of disseminating the knowledge gained to the larger community or industry.

#### Positive deviance approach

The positive deviance approach integrates some of the strengths of each of these approaches by combining intensive organizational-level examination using qualitative methods with the broader-scale statistical analysis possible with a large sample of organizations. The positive deviance approach allows for the explicit integration of real-life implementation issues and organizational context because it seeks to characterize not just what processes and practices are present in top performing organizations but also the context (*e.g*., organizational culture, leadership support, norms of behavior) in which they are implemented. These practices are characterized by extracting common themes or hypotheses based on several, rather than single, organizational settings where the proof of concept exists. This attention to organizational context is particularly important for complex, adaptive organizations [43] such as many health care organizations, which have multiple objectives and authority structures, and diverse technological underpinnings of their production functions. Although the replication of best practices requires sensitivity to the unique organizational context of the adopting organization [33,34,39,44-46], the positive deviance approach characterizes important contextual factors as part of the description of how top performers achieved their success.

In addition to the advantage of using scientific methods that address concerns of organizational context, the positive deviance approach also uses statistical analysis to develop evidence that supports or refutes the many hypotheses developed from the qualitative study. The combination of these methods identify practical solutions because they are by definition already implemented in some organizations, which are also robust in that they are supported by statistical evidence. For adopters, the presence of statistical information infers that the effectiveness of these practices in other organizations was not just by chance alone, but that their implementation is likely to result in improved performance in other organizations as well.

Despite these strengths of the positive deviance approach, there are limitations relative to the other approaches. In some but not all cases, positive deviance studies may rely on self-reports of organizational practices rather than procedures of a controlled trial, which may result in reporting bias, although established survey methods can be used to limit measurement error [47-49]. Additionally, for some insights found through a positive deviance approach, particularly related to organizational context (*e.g*., inter-group relations, power dynamics), it may be difficult to create valid, quantitative measures; in such cases, evidence may come solely from qualitative studies, which may not have credibility among certain individuals who are central to successful uptake and implementation. Furthermore, relative to quality improvement and action research efforts, the positive deviance approach focuses on organizations learning from external sources rather than internal process improvement efforts. Consequently, staff members of adopting organizations may not achieve the same level of learning and investment as they might if they were to develop best practices themselves. Nevertheless, even if the practice originates from outside the focal unit or organization, its adoption into a new organization typically requires adaptation to local circumstances in which staff must engage and hence learn [50]. Finally, characterizing best practices based on current performance may limit the expansive nature of discovery to what is achievable within the bounds of current constraints and approaches. Therefore, the positive deviance approach should be balanced with sustained *de novo *discovery efforts that periodically can fully shift the paradigm of an industry in ways not possible through the study of only positive deviance.

Ultimately, there are two major differences between the positive deviance approach and a quality improvement or action research approach. First, in positive deviance approaches, the best practices are assumed to already exist; they are not built *de novo *through a quality improvement of action research cycle of inquiry. Second, the source of best practices differs. Whereas quality improvement methods seek to discover through experimentation and data feedback within the organization, the positive deviance approach focuses on learning from exceptional examples of extant performance external to the focal unit or organization.

#### Dissemination of best practices

**Figure 2:**
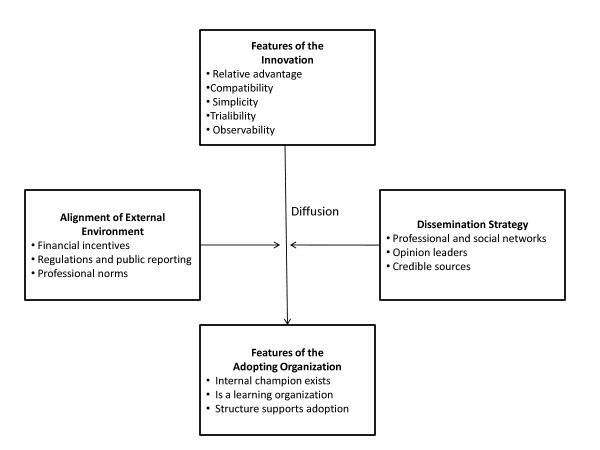
**Key drivers of the diffusion process**.

Promoting wide dissemination of best practices, particularly among health care organizations, has been the subject of expansive theoretical inquiry [45]. A distinguishing strength of the positive deviance approach is the focus on active dissemination of best practices. Existing theories [44,46,51-55] identify several factors that influence the shape of the trajectory of diffusion, or spread, of innovations throughout an industry (Figure 2): features of the innovation, the dissemination strategy, the alignment of the external environment with adoption of the innovation, and features of the adopting organizations, or users.

The positive deviance approach to identification and dissemination of best practices employs some of the key features thought to speed diffusion, or spread. First, some theoretical literature [44,45,52,53] suggests that innovations diffuse more quickly if they are perceived to provide advantage relative to the status quo, if they are compatible with current practices, if they are relatively simple to understand and implement, if they can be piloted, and if they generate observable improvements. Because data originate with top performing organizations in the positive deviance approach, best practices are largely viewed as providing relative advantage, being compatible with current practice (they are in place in some organizations already), and generating observable improvements (top performance can be measured). Second, the theoretical literature [45,52,53,56] also suggests the dominant mechanism for successful spread is interpersonal influence through professional and social networks, as well as links to opinion leaders. The credibility of communication channels both external to the organization and within the organizations are important. Critical to the positive deviance approach is that the top performers are those that have access to similar resources and come from the same communities or industry as potential adopters, allowing for greater interpersonal influence through existing professional associations and social networks [52,56] and engagement of opinions leaders, which is helpful to encourage initial adoption and subsequent implementation by users [45,52]. Finally, the positive deviance approach calls for the inclusion of potential adopters in the earliest studies of 'what works.' Organizational characteristics that make potentially adopting organizations and units within organizations more likely to adopt recommended changes are beyond the scope of the paper, and have been well-documented [44-46,51-55,57]; however, in the positive deviance approach, organizations participate closely in the research, and because the findings reflect their knowledge and experience, sites are often strongly motivated and receptive to implementing findings. Inclusion of stakeholders in producing relevant evidence for health care improvement has been shown to be successful in large-scale organizational changes [45,57].

### Using a positive deviance approach to improve care for acute myocardial infarction

#### Background

We used a positive deviance approach in our recent efforts to improve hospital care for patients with acute myocardial infarction. In the span of three years, the proportion of patients whose care met the targeted national guidelines for timeliness of care for ST-segment elevation myocardial infarction increased from about 50% to more than 75% of patients. The process reveals the potential of the positive deviance approach to identifying and disseminating best practices in order to accelerate whole-system change.

Prompt treatment is critical for survival of patients with ST-segment elevation myocardial infarction [58-60]. The time interval between symptom onset and hospital arrival, and between hospital arrival and treatment with percutaneous coronary intervention (PCI) (which can re-establish blocked blood flow to the heart) are important predictors of survival [59-61]. Although hospitals have less control over the time interval from symptom onset to hospital presentation, they have direct control over the time interval from hospital arrival to PCI, known as 'door-to-balloon time.'

As of 2004 to 2005, less than *one half *of patients received care that met the national target of door-to-balloon times within 90 minutes. Furthermore, performance had remained stagnant for several years with little improvement [62], despite substantial improvement in many other performance metrics for cardiac care [63]. Nevertheless, there were individual hospitals that were meeting the 90-minute guideline even before 2005 [64], thus illustrating positive deviance in this measure of quality of care.

### Positive deviance in action

#### Step one: Identify 'positive deviants,' *i.e*., organizations that consistently demonstrate exceptionally high performance in an area of interest

We used the National Registry of Myocardial Infarction [65], a patient registry of patients treated with primary PCI for acute myocardial infarction, to array participating US hospitals according to their median door-to-balloon times. From this list, we noted substantial variation in hospital performance across the industry. We identified the exceptional performers [66,67], those that had accomplished median door-to-balloon times of 90 minutes or less for their previous 50 cases. Within this group of approximately 35 hospitals, we ranked them by the degree to which they had improved in the previous four years, and selected from the hospitals with the greatest improvement. Using the resulting sample, we were able to examine what strategies were present at top performing organizations, circumstances prior to their top performance, and how the organization accomplished its improvements. We continued site selection with the same criteria until we achieved theoretical saturation [23,68], which occurred after 11 hospitals.

#### Step two. Study organizations in-depth using qualitative methods to generate hypotheses about practices that allow organizations to achieve top performance

We conducted in-depth site visits comprised of tours and open-ended interviews with all staff identified by the hospital as being involved with door-to-balloon time improvement efforts. This varied by hospitals but typically included cardiologists; emergency medicine physicians; nurses from the catheterization laboratory where PCI is performed; the emergency department; quality improvement units; technicians and technologists from various departments; emergency medical services staff, including ambulance staff; and senior and middle-level administrators. We interviewed a total of 122 staff members to understand their perspectives and experiences in improving door-to-balloon time at their hospitals. Researchers with diverse clinical and non-clinical backgrounds conducted the interviews in teams of two. After appropriate consent and institutional review board approval, interviews were audio-taped and transcribed by a professional, external transcription service. Interview teams underwent a formal debriefing with an organizational psychologist, and these sessions also were tape-recorded and summarized to identify possible additions to subsequent interviews and insights pertinent to the particular visit. All qualitative data, including the transcriptions of interviews and notes from the visit, were analyzed using the constant comparative method of qualitative data analysis [23,69,70]. This process was accomplished in teams of three to four individuals with differing backgrounds (*i.e*., clinical medicine, nursing, quality improvement, health services research, and management), including the two people who were present on the site visit as well as two researchers who participated in analysis of all data. Coded data were organized and further analyzed for recurrent and unifying themes using NUD*IST 4 (Sage Publications Software and now replaced by NVivo 8). We identified a set of specific strategies [66] that potentially were causally related to hospitals' improvement in door-to-balloon time. We also identified a number of characteristics of the organizational context [67] (such as senior management support, shared goals, physician leaders and interdisciplinary teams, data feedback, and ability to manage paradoxes) that we hypothesized were related to top performance.

#### Step three. Test hypotheses statistically in larger, representative samples of organizations

Based on hypotheses from the qualitative study, we developed a web-based hospital survey using closed-ended items. The sample comprised a randomly selected set of 365 hospitals that had treated at least 12 patients with primary PCI in the last year, and that participated in the National Registry of Myocardial Infarction. The survey was typically completed by a single individual who was requested most often to coordinate responses that represented an organization-wide response to the items. The respondent was typically the quality improvement director, although individuals varied by hospital. We deliberately focused in this quantitative survey on those items that could be objectively and reliably measured with closed-ended items. Complementing the web-based survey responses with data on hospital door-to-balloon times from Health Quality Alliance, we estimated a regression model to statistically test the hypotheses that had been generated in the qualitative study about hospital strategies most associated with reduced door-to-balloon times. We used hierarchical linear modeling to account for clustering of patients by hospital. Based on this quantitative analysis of a national set of hospitals, we identified a finite set of hospital strategies that were statistically associated with better door-to-balloon time. We also estimated the minutes saved with each of the identified strategies [71]. Hospital strategies significantly associated (p < 0.05) with lower door-to-balloon times and the minutes saved with each strategy were as follows: activation of the catheterization laboratory by emergency medicine physicians instead of cardiologists (eight minutes); using a single call to activate the catheterization team (14 minutes); activating the catheterization team based on pre-hospital electrocardiogram while the patient is still en route to the hospital (15 minutes); having the expected interval between page and arrival of staff in catheterization laboratory of 20 to 30 minutes versus longer (16 minutes); and having real-time feedback on door-to-balloon times for catheterization laboratory and emergency department staff (nine minutes). All variables were centered at their mean value; therefore the changes in minutes are relative to those of hospitals with an 'average' score on all other items [71]. The magnitude of saved minutes for each strategy was estimated by setting all other strategies equal to their average value in the data set. The synthesis of findings from the quantitative and qualitative studies identified six key strategies and several contextual factors that were linked with better door-to-balloon times.

#### Step four. Work in partnership with key stakeholders including potential adopters to disseminate the evidence about newly characterized best practices

Throughout the process of collecting qualitative and quantitative data, the research team and the American College of Cardiology (ACC) were in discussion about how best to disseminate the findings. The selected vehicle for dissemination was the door-to balloon (D2B) Alliance , a public campaign [72] supported by 38 professional associations and agencies committed to the single goal of having 75% of patients with ST-segment elevation myocardial infarctions treated with PCI to have door-to-balloon times of 90 minute or less. Using the communication channels of the state governors for the ACC, cardiologists and senior administrators working in hospitals across the US were approached about enrolling their hospitals in the D2B Alliance campaign. Enrollment required completing a web-based form in which the chief executive officer of the hospital committed to the D2B Alliance goal of reducing door-to-balloon time.

The D2B Alliance made available a change packet and toolkit, held webinars, published newsletters of success stories, facilitated workshops at the ACC and AHA annual meetings, and managed an online community. All of the activities were open regardless of enrollment status, although all hospitals that were formally enrolled completed a web-based survey at the time of enrollment and approximately one year later to evaluate their changes in strategies adopted and reported physician and management support for their quality improvement efforts.

Several features of the D2B Alliance were developed to be consistent with the theoretical literature on diffusion, or spread, of innovations [52]. In terms of the features of the innovation, the D2B Alliance selected practices from the literature that that were viewed as having relative advantage compared with current practice, were most compatible with organizational resources, that were simple to adopt, that were very observable, and that could be piloted in a trial-and-error approach. In terms of the dissemination strategy, the D2B Alliance collaborated with 38 professional associations and agencies that co-sponsored the effort. Involving the ACC governors in each state ensured the integration of opinion leaders in the process. The research papers supporting recommendations were published in credible venues, enhancing the perceived validity of the recommendations.

In terms of alignment with the external environment, the D2B Alliance efforts occurred in a broader environment that was also promoting improvements in door-to-balloon time. The Centers for Medicare & Medicaid Services was beginning to report hospital achievement of door-to-balloon times of 90 minutes or less and include modest financial incentives for meeting performance targets; the professional organizations responding to peer-reviewed literature of the clinical importance of door-to-balloon time were supportive of improvement efforts, and physicians seeking re-certification through the American Board of Internal Medicine could use participation in the D2B Alliance activities as evidence of their quality improvement efforts.

Ultimately approximately 1,000 of the 1,400 US hospitals that perform primary PCI enrolled with the D2B Alliance, a 70% penetration rate in the industry. Survey data indicate that there has been a significant increase since 2006 in the use of the recommended strategies among enrolled hospitals (unpublished data), and data from before and after the D2B Alliance show significant three-year improvement in door-to-balloon times [73]. Whereas only about one half of patients met this guideline in 2005, by 2008 about 75% of patients had door-to-balloon times within guidelines. Although the improvement has been industry-wide, patients treated in hospitals enrolled with the D2B Alliance for at least three months were significantly more likely than patients treated at non-enrolled hospitals to have door-to-balloon times that met guidelines (unpublished data). Such accomplishments suggest that what was once positive deviance is becoming standard practice, and illustrate the potential of the positive deviance approach for improving quality in health care.

## Conclusion

The positive deviance approach holds much promise for improving practice. It takes advantage of natural variation in performance, develops an evidence base through detailed organizational analysis and statistical testing of hypotheses, and supports collaboration between researcher and practitioner in ways that identify feasible solutions and foster support for dissemination and uptake of recommendations. Practitioners and organizations can take advantage of positive deviance by identifying top performance within units of the organization or in other organizations, and foster examination and discussion of such performance in order to elevate performance in other areas. Barriers to its use may include competition between units within a single organization or between organizations such that secrets of success are not readily shared, structural separation of units so that information does not flow easily, or workforce issues in that employees do not see others' experience as adequately relevant to their own.

The case study illustrates the key steps to applying positive deviance methodology to improving hospital care for myocardial infarction and also highlights circumstances in which the positive deviance method may be most useful. First, in the case of door-to-balloon time, there was a concrete and widely-endorsed indicator of organizational performance. Second, the indicator could be assessed reliably for multiple organizations using existing data from national registries of patients with acute myocardial infarction and the national public reporting system for hospital quality. Third, substantial variation in hospital performance was apparent, with some exceptional performers but many that did not meet national guidelines. Fourth, organizations were willing to share their experiences openly to help produce needed evidence for how to improve performance. Finally, there was substantial impetus from both clinical and management staff to reduce door-to-balloon time. Reducing door-to-balloon times both benefited patient survival and enhanced organizational standing in a competitive, profitable market for which hospital performance was publicly reported. Together, these features created an ideal opportunity for using the positive deviance approach to identify and disseminate innovations to improve quality of care.

The gap between what we know and what we do is well-documented [39,74]. This gap is particularly pertinent in health care organizations, as the research literature on best medical practices is robust; however, findings are often not implemented reliably [37,39,75]. Researchers lament the limited adoption rates of best practice identified through research, and practitioners lament that the research is experimentally-based and hence not applicable to their daily practices. To bridge this gap between what we know and what we do, between research and practice, we suggest leveraging the naturally-occurring positive deviance to both identify best practices in ways that are robust, credible, and to promote widespread uptake of innovations in health care organizations.

## Competing interests

The authors declare that they have no competing interests.

## Authors' contributions

EHB is the lead author and the corresponding author of the paper. LAC, SR, LR, IMN, and HMK co-wrote the paper and have approved of the final draft of the manuscript.
